# Use of non‐invasive optical imaging techniques in dermatology

**DOI:** 10.1111/ddg.15980

**Published:** 2025-11-14

**Authors:** Deborah Winkler, Tamara Eyssele, Amelie Glanzer, Hanna Wirsching, Elisabeth Klein, Julia Welzel, Sandra Schuh

**Affiliations:** ^1^ Department of Dermatology and Allergology University Hospital Augsburg Augsburg Germany

## Abstract

Noninvasive imaging of skin diseases is highly valued in both dermatological research and practice. Reflectance confocal microscopy (RCM) is primarily applied to assess melanocytic lesions because of its high resolution, whereas optical coherence tomography (OCT) is particularly used for non‐melanocytic skin tumors owing to its greater penetration depth. Line‐field confocal optical coherence tomography (LC‐OCT) combines the techniques of reflectance confocal microscopy (RCM) and optical coherence tomography (OCT), enabling visualization of the skin down to the superficial dermis while simultaneously providing cellular resolution. The visualization of the skin changes can be done horizontally, vertically, in 3D mode or as a video in real time. The evaluation of the measurement can be supported by artificial intelligence if required. The field of application of LC‐OCT includes both melanocytic and non‐melanocytic lesions. In addition, preliminary studies show great potential in the diagnosis and therapy control of inflammatory and infectious skin diseases. Nevertheless, RCM and OCT still have their place in special cases such as tumors requiring a particularly high resolution or penetration depth > 500 µm.

## INTRODUCTION

In many cases, biopsy is the gold standard for excluding or confirming a suspected dermatological diagnosis. Apart from pain and scar formation in potentially sensitive areas, the invasive procedure has additional disadvantages: The duration of the histological diagnostic workup results in a delay for patients and physicians and may require another visit. Given that examination of larger lesions, such as lentigo maligna, may only be possible selectively and not completely, the risk of misdiagnosis exists. Moreover, the dynamics of skin changes can no longer be observed after excision. Accordingly, assessment of potential residual tumor portions during the disease course may be more difficult. Noninvasive imaging allows for diagnosis of many skin diseases without the disadvantages of biopsy. Reflectance confocal microscopy (RCM), particularly suitable for differentiation of melanocytic lesions, and optical coherence tomography (OCT), used especially for epithelial tumors, have been of major importance in daily clinical routine for many years.^1^ While RCM is characterized by a very high resolution of approximately 1 µm and a low penetration depth (approximately 300 µm), OCT has a lower resolution of approximately 7.5 µm and a high penetration depth of up to 1.5 mm.^2^ The development of line‐field confocal optical coherence tomography (LC‐OCT) has combined the advantages of high resolution (1–2 µm) and good penetration depth (500 µm), thus enabling the assessment of both melanocytic and non‐melanocytic tumors at the cellular level.^3^ Moreover, the spectrum of clinical fields of application is constantly expanding from skin tumors via infectious to inflammatory diseases.

### In vivo reflectance confocal microscopy

#### Operating principle and technical details

Reflectance confocal microscopy (RCM) is a noninvasive imaging method providing images of almost histopathological accuracy.^4^, ^5^ In vivo images of skin lesions can be generated in real time to answer questions concerning differential diagnosis and assess diseases over time.^5^ This technique relies on a laser emitting light at a wavelength of 830 nm.^5^ The laser beam is directed to a single point in the skin and the light in this point is reflected at interfaces with a high refractive index.^6^ A detector processes exclusively the reflection of the point in focus, given that scattered light of surrounding tissue will not penetrate through the pinhole aperture.^4^, ^6^ By scanning an area of the skin, the instrument generates an overall picture based on the different refraction indices of individual cells and tissue components.^4^, ^6^ This results in a gray‐coded image showing cell structures and microstructures on a grayscale depending on the reflection of light.^4^, ^6^ The VivaScope 1500/3000 (VivaScope GmbH, Munich, Germany) has a penetration depth of up to 150–300 µm permitting the visualization of epidermis and upper dermis (papillary and upper reticular dermis).^6^ Eventually, horizontal *en‐face* images are generated showing a certain layer of the skin.^6^ By producing scans in different planes of the skin (*stacks*) and lateral fusion of the individual images (mosaic), this results in comprehensive illustration of the lesion.^4^, ^6^ With this technique, RCM allows for a high resolution at the cellular level of up to 1 µm and visualization of cells, such as keratinocytes, melanocytes, and cellular components like melanin.^5^ Currently available devices include the static (VivaScope 1500; VivaScope GmbH) and the mobile, handheld RCM instrument (VivaScope 3000; VivaScope GmbH).^7^

#### Clinical application

RCM has been utilized for more than 30 years to differentiate, analyze, and observe skin changes In vivo.^6^, ^8^ RCM plays a crucial role in differentiation of malignant and non‐malignant melanocytic skin lesions.^8^ Based on certain criteria, it is possible to determine whether a lesion is a malignant melanoma or a benign nevus.^9^ Criteria for making a diagnosis are outlined in the examples given below. In addition, this noninvasive imaging method enables the differentiation of non‐melanocytic lesions from each other.^6^ In an article from 2015 analyzing 100 skin tumors by RCM, the authors reported a specificity of 72%–80% and a sensitivity of up to 89%–100% with respect to the correct diagnosis.^8^ RCM enables also the assessment of tumor size and margins of a skin tumor.^10^ This will, for example, permit verification that a lentigo maligna has been excised completely or whether re‐excision is required.^10^ Moreover, RCM is suitable for monitoring the therapeutic response in, for example, actinic keratosis.^5^ This aspect plays also a role in other diseases, such as vitiligo, psoriasis, and alopecia.^4^, ^11^ Again, RCM facilitates diagnosis of these diseases and permits the evaluation and, if indicated, change of the therapy.^12^, ^13^

#### Case studies

In order to understand the characteristics of different diseases in RCM, it is important to know the features of healthy skin. Gonzales et al. have described the findings of healthy skin already in 2003.^6^ First, the already mentioned orientation in *en‐face* view and the black and white image of the skin need to be considered.^6^ Beginning with the stratum corneum, the images show a very bright, almost continuous white layer.^6^ The grouped corneocytes without nuclei are only separated by dark lines created by skin creases. This is followed by the stratum granulosum.^6^ Here, the cells have a dark nucleus surrounded by bright, granular cytoplasm. The morphology of the next layer is described as honeycomb pattern, given that the cells in the stratum spinosum resemble honeycombs.^6^ The cells are somewhat smaller and the cell borders are more distinct than in the stratum granulosum. With increasing penetration depth, suprapapillary epidermis and dermoepidermal junction (DEJ) become finally apparent.^6^ In this area, the keratinocytes are smaller and very bright due to the high melanin content.^5^ The melanin is usually arranged supranuclearly, resulting in a picture of *melanin caps* or *umbrellas*.^5^ Moreover, the melanocytes present as bright, solitary cells with round, oval, fusiform, or dendritic morphology.^5^ The basal cells present as bright rings surrounding the papillary tips. Blood vessels are often identified in the center of the papillae. In the adjacent superficial papillary dermis, blood vessels and reticular fibers are observed, too. The findings of healthy skin in RCM may vary individually based on skin type, sun exposure, and skin thickness.^6^

**FIGURE 1:**
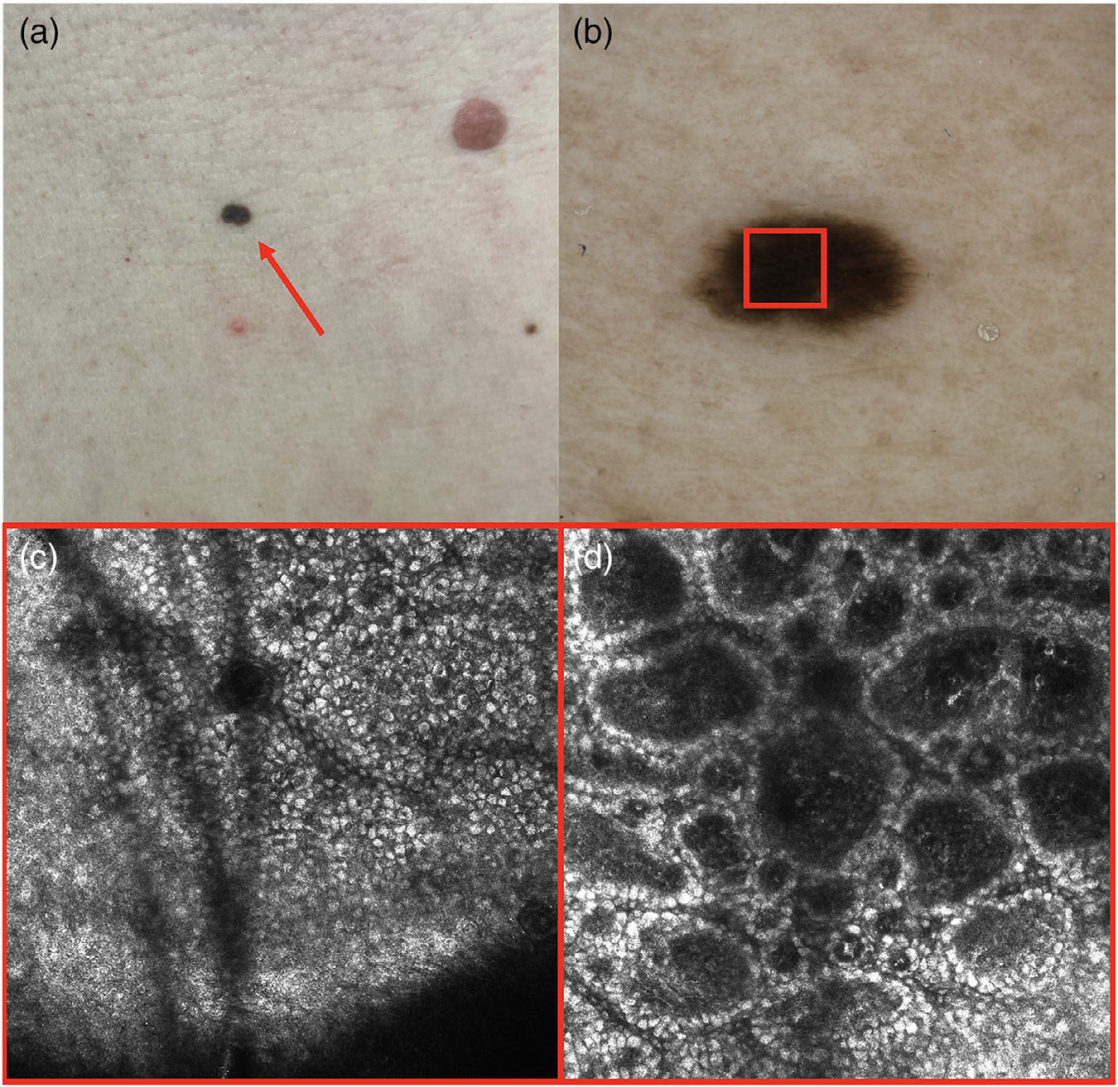
(a) A compound nevus on the abdomen, shown clinically (red arrow). (b) Dermoscopic image. (c) Reflectance confocal microscopy image (RCM, VivaScope 3000^®^, VivaScope GmbH, Munich, Germany, image size 0.5 × 0.5 mm) representing the red square marked in the dermoscopic image (b): cobblestone pattern in the epidermis. (d) Image at the level of the dermoepidermal junction and in the upper dermis: regular dark papillae embedded in a ring‐shaped pattern of bright basal keratinocytes. Depicted is a regular junctional nevus.

**FIGURE 2:**
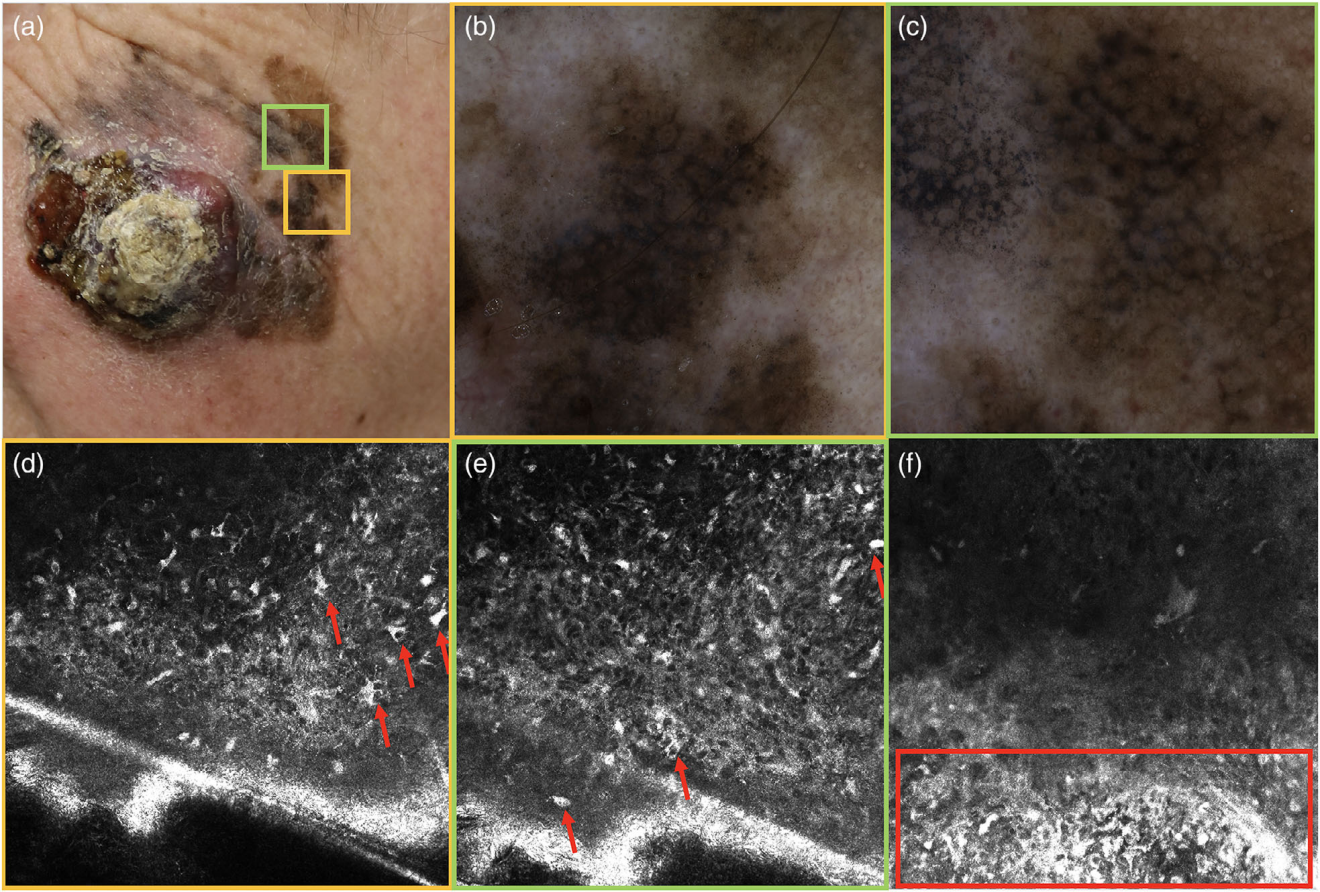
(a) Nodular melanoma, tumor thickness > 1.7 cm, left cheek. (b) Dermoscopic image of the yellow‐marked area in (a). (c) Dermoscopic image of the green‐marked area in (a). These sections show asymmetrically pigmented follicular openings, rhomboid structures, gray‐black dots, an irregular structure, and various colors ranging from light brown to dark brown‐gray to black. Furthermore, an annular granular pattern with gray rings is visible. (d) Reflectance confocal microscopy (RCM, VivaScope 3000^®^, VivaScope GmbH, Munich, Germany, image size 0.5 × 0.5 mm) of the yellow‐marked area in (a): no typical honeycomb or cobblestone pattern in the epidermis, but numerous ascending pagetoid cells (red arrows). (e, f) RCM images of the green‐marked area in (a): numerous round pagetoid cells and a chaotic organization are evident (red box).

In RCM, the melanin of the skin, in particular, appears bright, thus generating a strong contrast.^5^ Melanocytes and their pathological changes can also be visualized in RCM.^6^ Consequently, melanocytic lesions or diseases resulting in altered skin pigmentation are particularly suited for assessment by RCM. In the following, a melanoma examined by RCM in comparison with a melanocytic nevus is analyzed as an example (Figures 1, 2). Pellacani et al. identified six criteria correlating most strongly with malignity. These characteristics include round, large pagetoid cells, non‐edged papillae in the epidermis, epidermal disorganization of cells (absence of honeycomb or cobblestone pattern), nuclear cells in papillary dermis, pleomorphic pagetoid infiltration, and absence of junctional cell nests.^9^ In contrast, melanocytic nevi showed a regular honeycomb or cobblestone pattern in the epidermis, regular papillae at the level of DEJ, and dense sparse homogeneous nests/clods, each embedded in a ring‐like pattern, in the upper dermis (Figure 1).^9^

In RCM, presence of large, pagetoid, or dendritic cells, loss of the regular honeycomb pattern, and presence of irregular nests of atypical melanocytes are indicative of malignant melanoma.

**FIGURE 3:**
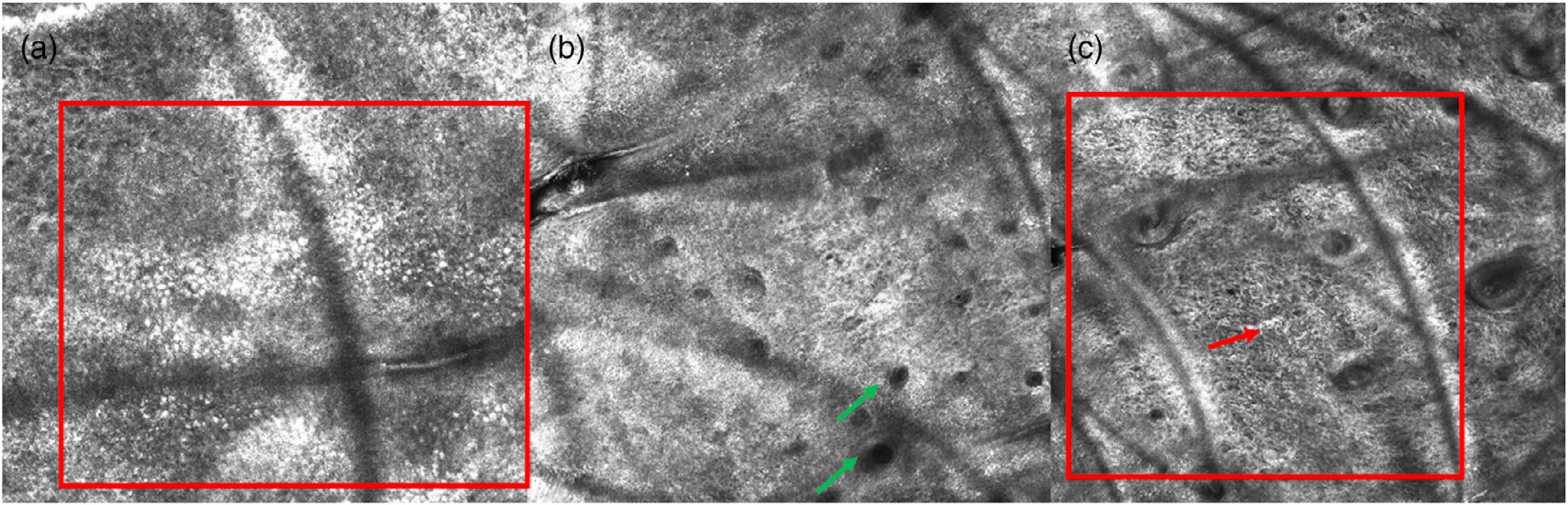
(a) Epidermis in vitiligo imaged by reflectance confocal microscopy (RCM, VivaScope 3000^®^, VivaScope GmbH, Munich, Germany, image size 0.5 × 0.5 mm). Successful treatment reveals pigmented keratinocytes resembling a cobblestone pattern (red square). (b) Dermoepidermal junction (DEJ): empty papillary tips (green arrows). (c) DEJ and papillary dermis: large dendritic melanocytes (red arrow and red square) and the first pigment rings in the basal layer as a sign of repigmentation.

The autoimmune disease vitiligo is another disease where RCM is particularly important for therapy monitoring. Goal of the therapy is repigmentation of affected skin areas. In vitiligo, active and stable disease phases are distinguished. Cortelazzi et al. described that the lesions show a complete loss of melanin in the active disease phase.^12^ Moreover, in active lesions, RCM depicts no bright keratinocytes or melanocytes in the epidermis.^11^ At the level of DEJ, a loss of the normal ring structure is observed. Only “shadows” of the dermal papillae are identified.^11^ It should be emphasized that in this phase the non‐affected skin of patients with vitiligo shows also differences compared to findings of healthy individuals.^11^ Compared to healthy individuals, the characteristic ring structure at the level of the junction zone is also difficult to identify in the perilesional area. Incomplete distribution of cells around the papillae results in a picture of “half rings” or, if these are connected, in structures with “arc‐shaped rims”.^12^ This results in ill‐defined lesion borders that are difficult to assess. Sometimes, residuals of individual dendritic melanocytes can be observed in perilesional areas of the skin.^11^ In addition, bright inflammatory cells are depicted in the papillary dermis.^13^ Compared to RCM images of healthy individuals, this results in a different pattern in patients with vitiligo with respect to image contrasts and brightness.^11^ In the stable phase of the disease, no changes with respect to melanin content or papillary ring structure are observed in non‐affected skin areas.^12^ The border between healthy and diseased skin is clearly identified.^13^ RCM is suitable for assessing whether the therapy results in improvement corresponding to repigmentation.^13^ Repigmentation is indicated by activated melanocytes with bipolar, star‐like, or dendritic morphology along the DEJ.^12^ This is also confirmed by an increase of pigment granules.^13^ Infiltration of melanocytes may follow various patterns: starting from the adjacent healthy skin, from the hair follicles, or diffusely within the lesion.^12^ In addition, response to therapy is also corroborated by pigment rings in the basal layer (Figure 3).^13^

#### Advantages and limitations

RCM offers numerous advantages. The reliable diagnosis without biopsy should be emphasized, in particular. In combination with dermoscopy, RCM shows an increased sensitivity for malignant skin changes.^14^ It is the aim to perform excisions only after In vivo confirmation of a malignant lesion in order to avoid overtreatment of benign lesions. This is a gentler approach for patients, given that invasive interventions are performed only in case of a respective indication.^6^ Especially in areas with a high risk of cosmetically disfiguring scars, this allows for a more specific treatment.^4^ Moreover, the lower number of biopsies and histological examinations will increase efficiency in clinical routine while the time between diagnosis and therapy initiation will be shorter.^4^, ^7^ In addition, the immediate determination of tumor margins enables complete excision and reduces re‐excisions.^10^ RCM shows its potential also in therapy monitoring, especially in superficial skin tumors with topical treatment.^6^, ^7^ The disease course can be visualized repeatedly, noninvasively, and in real time, thus improving the therapy management in chronic diseases.^11^ Moreover, it allows for external or telemedical image assessment increasing objectivity and reliability.^15^ In terms of perspective, image interpretation might be supported by AI‐based programs. Compared to other In vivo methods, RCM is convincing with its high resolution and image quality, permitting precise assessment of pigmented lesions.^8^

These advantages are, however, opposed by some limitations. Due to the low penetration depth, deeper tumors cannot be visualized completely, especially in case of hyperkeratosis.^5^ Accordingly, the infiltration depth must be determined with other procedures. In addition, lesions in difficult‐to‐access areas – such as in the nasolabial fold or ear – are difficult to analyze with the static RCM device.^6^ Horizontal sections impede the interpretation compared to vertical depth sections familiar from histology. Extensive training is required for reliable evaluation resulting in interindividual differences in the diagnosis.^15^ It has, however, been shown that additional telemedical assessment by experts will increase the sensitivity.^15^ Nowadays, many diagnostic features are well described and first diagnostic algorithms exist.^16^ Nevertheless, histology remains the gold standard. Another limiting factor is the restricted availability of the instruments – usually only in specialized centers. Reasons are the high acquisition costs and the higher time expenditure per examination compared to dermoscopy.^7^ This impedes its integration in clinical routine. As a solution, Levine et al. proposed pre‐screening of suspicious lesions with dermoscopy to utilize RCM predominantly in lesions difficult to assess.^7^ Improvements in AI and other technical fields might significantly expand the use of RCM.

Despite existing limitations, RCM offers a high potential – for both patients and dermatologists. However, further optimizations are required to make the method more feasible in daily routine.

Based on its high resolution at the cellular level, reflectance confocal microscopy is particularly suited for assessing the potential malignity of melanocytic lesions. Due to the low penetration depth, it is usually not possible to draw conclusions about the invasion depth of tumors.

### (Dynamic) optical coherence tomography

#### Operating principle and technical details

Optical coherence tomography (OCT) is a noninvasive imaging method providing high‐resolution cross‐sectional and *en‐face* images of various biological tissues.^17^ Originally developed for examination of the human eye, it is now used in various medical disciplines including dermatology. OCT is based on the principle of interferometry, especially Michelson interferometry, which uses light with short coherence length (wavelength between 700 nm and 1,300 nm).^1^, ^2^ In this technique, light reflected from tissue is used for reconstruction of two‐ and three‐dimensional presentations of skin tissue.^1^, ^2^, ^17^ As imaging method, OCT falls between high‐frequency ultrasound and RCM with respect to resolution and imaging depth (1.5 mm).^17^ Furthermore, the development of “dynamic optical coherence tomography” (D‐OCT) allowed for visualization of the morphology of blood vessels in addition to tissue structures. This technique is based on the recognition of motile particles in OCT images: Images are acquired with a high frame rate and subsequently analyzed for changes. This allows for differentiation of moving particles corresponding to blood flow from the static tissue signal.^18^ Given that the skin is easily accessible and many skin diseases are superficial, OCT is an ideal diagnostic tool in dermatology.

#### Clinical application

In the last 30 years, OCT has established itself as a valuable noninvasive imaging technique in dermatology. It permits high‐resolution imaging of the skin in real time with visualization of skin layers. OCT has demonstrated its worth in diagnosis and differentiation of keratinocyte skin cancer, especially basal cell carcinoma (BCC), squamous cell carcinoma (SCC), and actinic keratosis (AK).^17^, ^19^ In addition, it enables assessment of skin infections, inflammatory diseases like psoriasis, measurement of skin thickness and structural changes, or the analysis of skin vasculature by means of D‐OCT. Below, diagnostic criteria are elaborated in case studies of BCC and AK. In trials, sensitivity and specificity of OCT in BCC range between 79% and 94% and between 85% and 96%, respectively.^1^, ^18^, ^26^ In combination with dermoscopy and medical experience, it is a valuable tool for early diagnosis. Moreover, OCT may contribute to determination of tumor thickness and lateral extension – decisive factors for selecting the optimal therapy. While excision is the first‐line therapy in BCC,^20^ superficial tumors (≤ 1.2 mm) may also be treated topically with imiquimod, 5‐fluorouracil, or photodynamic therapy (PDT). With a penetration depth of 1.5 mm, OCT enables reliable detection of BCC down to a depth of at least one millimeter and to make an appropriate therapeutic decision.^21^ In addition, determination of tumor margins with OCT may increase the precision during removal. This is particularly beneficial in Moh's micrographic surgery: OCT permits precise In vivo differentiation of tumor margins and may thus help to avoid unnecessary surgical interventions or facilitate complete resections.^22^ Intraoperatively, OCT assists in identification of residual tumor at excision margins, which may reduce the number of additional resections.^23^ Wound management is another field of application of (D)‐OCT: Compared to traditional histological analysis, acute and chronic wounds can be monitored repeatedly and without tissue collection with (D)‐OCT. Morphological changes during the healing process, especially in epidermis and dermis, can be analyzed without taking biopsies. In particular, observations of vascular changes facilitate early recognition of disturbed wound healing or differentiation of ulcers of different etiology.^24^ Furthermore, OCT has proven to be a valuable tool in diagnosis and monitoring of inflammatory and blistering skin diseases, such as psoriasis, contact dermatitis, or bullous pemphigoid.^17^, ^18^, ^25^, ^47^ In addition, OCT can be used for monitoring the dynamics of skin changes during treatment. For example, it has been demonstrated that it can recognize the decrease of epidermal thickness caused by steroids at an early stage, which may enable better monitoring of the therapy while also avoiding side effects.^17^

#### Case studies

First, an explanation concerning the features of healthy skin in OCT is required in order to understand the diagnostic criteria of BCC and AK. The stratum corneum presents as thin hyperreflective band at the top of the image. In horny skin, for example, on the palms, it is visible as dense, homogenous structure. The epidermis presents as a dark, homogenous layer while the dermis is shown as a brighter region of higher signal intensity with areas of low signal intensity (for example, blood vessels and hair follicles). The DEJ separates the epidermis from the dermis and is often visible as a fine, hyporeflective line.^26^

Characteristics and differences of superficial, nodular, and infiltrative basal cell carcinomas are depicted in Table 1.^27^

**TABLE 1:** Characteristic features and differences of the three subtypes of basal cell carcinoma (BCC) – nodular, superficial, and infiltrative – according to Fuchs et al.^27^.

CHARACTERISTIC	NODULAR BCC (NBCC)	SUPERFICIAL BCC (SBCC)	INFILTRATIVE BCC (IBCC)
GROWTH PATTERN	Nodular, well‐defined	Superficial, bound to epidermis	Deeply infiltrating, aggressive
TUMOR NESTS	Large, round hyporeflective tumor nests	Connected to epidermis, lobular, hyporeflective	Small, dense tumor islands
CONNECTION TO EPIDERMIS	Partly connected	Strong connection with interruption DEJ	Usually separated
EPIDERMAL CHANGES	Focal atrophy	Thickened epidermis, irregular	Minor epidermal involvement
PERITUMORAL STROMA	Hyperreflective stroma with hyporeflective rim and halo (peritumoral cleft formation)	Hyporeflective demarcation and cleft formation	Hyperreflective stroma (fibrosis)

**FIGURE 4:**
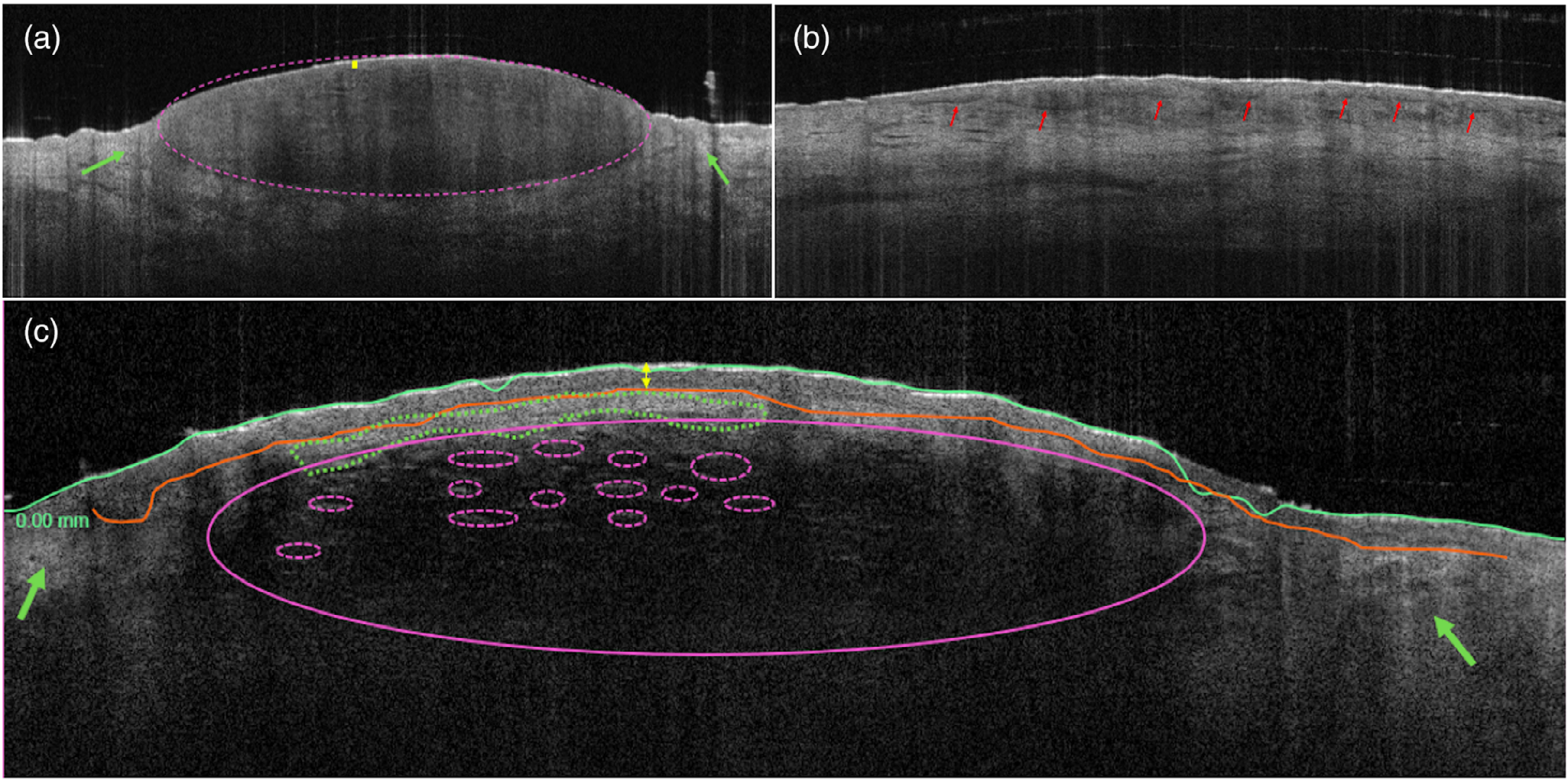
(a) Nodular basal cell carcinoma (BCC) visualized by optical coherence tomography (OCT, VivoSight Dx, Michelson Diagnostics, Kent, United Kingdom; horizontal image size 6 × 2 mm): purple oval = nodular, hyporeflective tumor nodule; yellow line = epidermis; green arrow = hyperreflective surrounding stroma. (b) Superficial BCC: red arrows = epidermis‐bound basal cell carcinoma nests. (c) Invasive BCC: yellow double‐headed arrow = epidermis; purple ovals = deeply infiltrating BCC with many small, dense tumor islands (dashed); green dashed area and green arrows = hyperreflective stroma; green solid line = automatic detection of the skin surface by the OCT device.

Another study described the absence of hair follicles and glands in addition to the criteria already mentioned.^28^ In Figure 4, the criteria of Fuchs et al. and the criteria mentioned in other studies are illustrated.^27^

**FIGURE 5:**
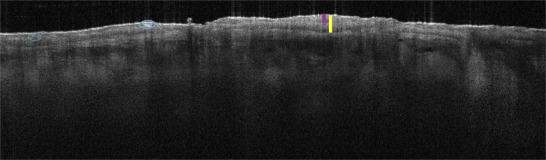
(a) Actinic keratosis imaged by optical coherence tomography (OCT, VivoSight Dx, Michelson Diagnostics, Kent, United Kingdom; horizontal image size 6 × 2 mm): purple line = thickened stratum corneum; yellow line = thickened epidermis; light blue ovals = hyperreflective areas due to keratin deposits.

The diagnostic criteria of AK are diverse. AK presents with disruption of skin architecture and DEJ in OCT. The epidermis appears irregularly thickened, often in association with hyperkeratosis (dark, broadened stratum corneum) and white streaks or dots arising from keratin deposits and visible as hyperreflective areas.^26^, ^28^ Figure 5 illustrates the mentioned criteria of AK.

#### Advantages and limitations

OCT has established itself as noninvasive imaging method with several strengths. It enables the painless examination of skin without requirement of a biopsy. Especially in diseases like BCC, OCT images are consistent with the results of histopathological examination.^29^ This is beneficial in patients with multiple skin lesions or if the number of tissue biopsies should be reduced for aesthetic or medical reasons.^30^ A significant advantage is the diagnosis in real time. Given the immediate availability of images, informed therapeutic decisions can be made dependent on clinical and patient‐related characteristics.^21^ In addition, OCT is useful for presurgical determination of tumor margins – especially in Moh's micrographic surgery – given that it supports targeted excision of the tumor with maximum sparing of healthy tissue and reduces the number of required tissue sections.^29^, ^31^ Moreover, OCT is suited for follow‐up and therapy monitoring, for example, in photodynamic therapy or topical application of imiquimod. Regular OCT controls reveal whether the therapy is successful or requires adjustment.^32^ Despite these benefits, OCT has also limitations. Given its penetration depth of 1.5 mm, complete assessment of deeper skin structures or invasive tumor portions – for example, in nodular or infiltrative basal cell carcinomas – is not possible.^31^ The so‐called “iceberg phenomenon” describes the potential underestimation of the measured tumor extension in terms of depth.^23^ Moreover, the cellular morphology in OCT images is less detailed than in histopathological tissue samples. Although the architecture of many lesions is recognized in OCT and can be used for diagnosis, atypical melanocytes cannot be differentiated. Therefore, biopsies are still required.^33^ Moreover, melanomas cannot be differentiated reliably from benign nevi by their structural features.^34^ Accordingly, biopsy and histopathological examination remain the gold standard for the final diagnosis. However, D‐OCT can provide evidence concerning the differentiation of nevi and melanomas by analyzing their microvasculature.^34^

A significant advantage is the diagnosis in real time. Given the immediate availability of images, informed therapeutic decisions can be made dependent on clinical and patient‐related characteristics.

Although the architecture of many lesions is recognized in OCT and can be used for diagnosis, atypical melanocytes cannot be differentiated. Therefore, biopsies are still required.

The combination of RCM and OCT is increasingly gaining importance. It enables detailed evaluation of dermatological diseases while improving presurgical planning concerning risk assessment and monitoring. It may help in increasing diagnostic accuracy and in avoiding biopsies.^35^ However, quality and interpretation of OCT images are highly dependent on the experience of the investigator,^28^, ^30^ while a standardized diagnostic workup is not yet available. Apart from these limitations, there are also practical challenges, such as high acquisition costs and limited availability. Overall, OCT is a promising, fast, and painless method for assessment of keratinocyte skin tumors, inflammatory skin diseases, and therapy control. However, its limitations, such as low penetration depth and restricted specificity in certain lesions, still exist – histology remains indispensable in these areas.^36^

Optical coherence tomography is primarily used for the differentiation of epithelial tumors, especially basal cell carcinomas. For this purpose, the different growth patterns (superficial, nodular, infiltrative growth) and the invasion depth up to a tumor thickness of 2 mm can be determined and used for therapeutic decisions.

### *Line‐field* confocal optical coherence tomography

#### Operating principle and technical details

*Line‐field* confocal optical coherence tomography (LC‐OCT) was introduced as advancement of optical coherence tomography (OCT) in 2018. It combines the principles of OCT with RCM and enables measurements with cellular resolution and medium penetration depth in real time. The tissue can be visualized in horizontal and vertical planes as well as in 3D mode.^3^ Using a two‐beam interference microscope with a laser source of 800 nm wavelength, the skin surface is scanned down to a depth of 500 µm and reflected and scattered light is collected.^37^ Depending on its intensity, the reflected light is visualized on the screen in different shades of gray. Skin structures with high refractive index, for example, strongly pigmented cells or keratin in stratum corneum, appear rather bright due to the high reflection of the light beam, while water‐containing structures appear dark. Currently, the only LC‐OCT instrument commercially available is deepLive^TM^ from DAMAE Medical (Paris, France). The lateral and axial resolution of 1.3 µm and 1.1 µm, respectively, is comparable to RCM. While the penetration depth of 500 µm is lower compared to OCT (1,500 µm), it is usually sufficient for assessing epidermis and superficial to mid‐dermis. Images can be visualized via three different modes in real time. The image size corresponds to 1.2 x 0.5 mm^2^. The view in horizontal mode (*en‐face*) is similar to RCM. Due to the high resolution at the cellular level, the view in vertical mode (*en‐coupe*) is similar to a histopathological section. As third mode, a 3D video reconstruction of the tissue can be generated by means of an auxiliary program (3D Slicer, TheSlicer Community, open‐source software). The device is equipped with a CE‐certified software reporting the probability for the presence of basal cell carcinoma, actinic keratosis or squamous cell carcinoma, seborrheic keratosis, dermal nevus, or another melanocytic lesion in percent. In addition, deepLive^TM^ enables visualization of blood flow and route of blood vessels. Parallel to the LC‐OCT presentation, the measured area is depicted on the screen by dermoscopy to improve the orientation and stored. The field of view has a diameter of 2.6 mm and a resolution of 5 µm.^38^ Given that dermoscopic and LC‐OCT images are synchronized by the colocalization software, dermoscopic characteristics can be compared simultaneously.^38^ In addition, the software can fuse adjacent areas by means of a mosaic reconstruction procedure to one large image. Both individual pictures and videos can be stored for documentation. This enables assessment of the measurement both in real time and retrospectively. In practical implementation, several drops of immersion oil are required between skin contact and probe. It should be taken into account that certain locations, for example, the inner canthus, are difficult to access due to the size of the handpiece. Individual images are acquired nearly in real time while the complete three‐dimensional measurement usually takes less than one minute.

The assessment of measurements acquired by LC‐OCT can be supported by CE‐certified artificial intelligence in real time.

#### Clinical application

**FIGURE 6:**
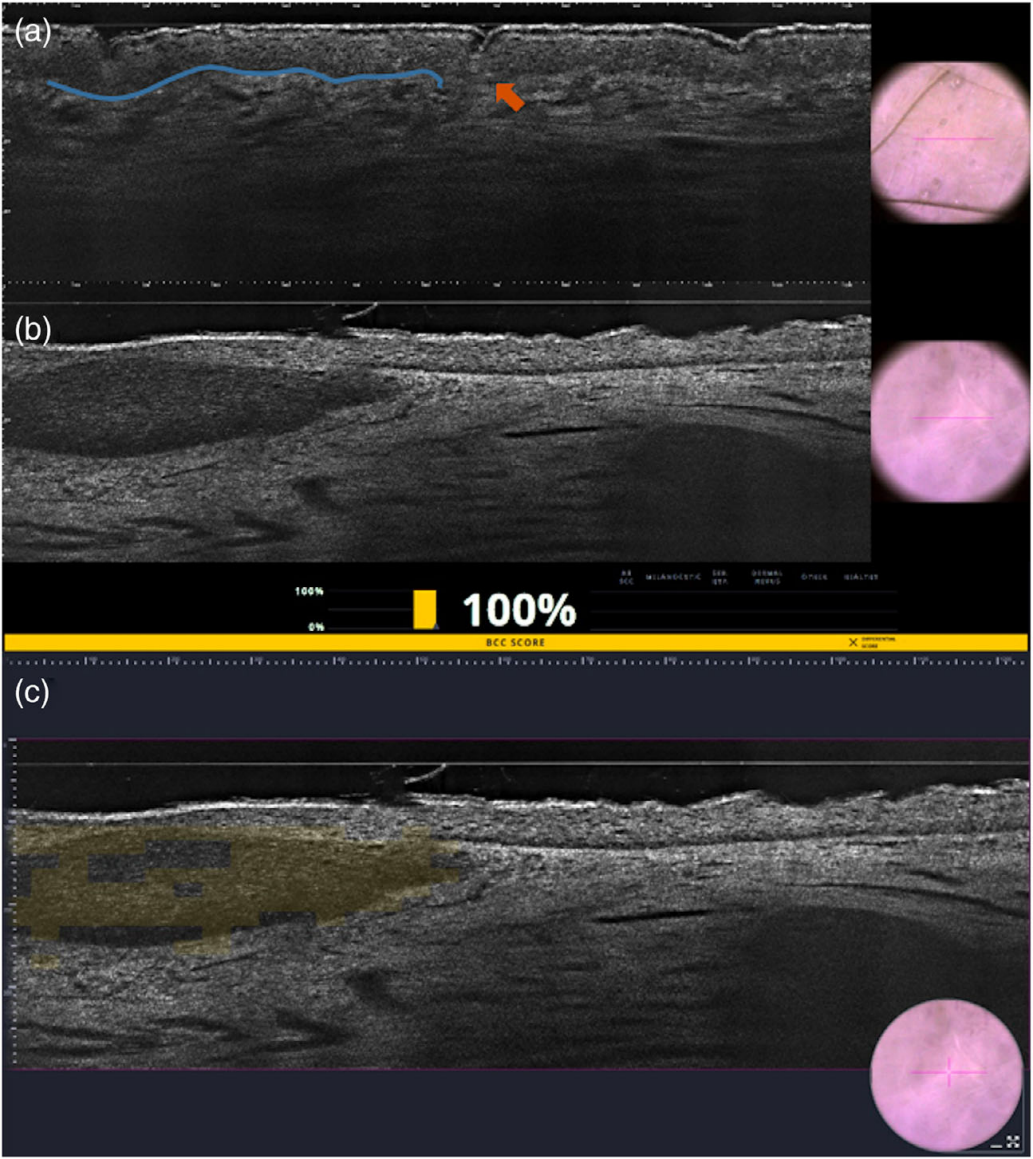
Visualization of various findings in the vertical view using line‐field confocal optical coherence tomography (LC‐OCT, deepLive™, DAMAE Medical, Paris, France, horizontal image size 1.2 × 0.5 mm). Simultaneous dermoscopy is visible on the right side of the image. (a) Healthy skin: various layers of the epidermis, the dermoepidermal junction (blue line), and adnexal structures such as hair follicles (orange arrow) are visible down to the papillary dermis. (b) Superficial basal cell carcinoma: on the left side of the image, an ovoid, hyporeflective tumor cone with peritumoral cleft formation is visible. (c) Superficial basal cell carcinoma with AI‐assisted assessment (identical lesion as in (b)): the superficial basal cell carcinoma detected by artificial intelligence is hatched in yellow. The AI indicates a diagnostic certainty of 100%.

The fields of application of LC‐OCT comprise a wide spectrum from malignancies to inflammatory and infectious skin diseases. Previous experience in dermatohistology or OCT is an advantage for learning the interpretation of LC‐OCT images. Apart from the layers of the epidermis, DEJ and adnexal structures, such as hair follicles or ducts of sweat glands, can be visualized at the cellular level down to the papillary dermis. So far, the best‐studied applications of LC‐OCT are diagnosis and subtyping of basal cell carcinomas (Figure 6).^39^ While superficial or nodular basal cell carcinomas often present with bud‐like or ovoid, hyporeflective tumor nests with dark rim (clefting) originating from the epidermis, basal cell carcinomas of the infiltrative type present with narrow shoal of fish‐like tumor strands.^40^ In this context, LC‐OCT is not only useful for primary diagnosis, but also for therapy monitoring of patients on topical therapy, laser treatment, or to define a surgical area.^41^, ^42^ In addition, LC‐OCT is suitable for detection of actinic keratoses and for their categorization based on PRO classification.^43^ In invasive squamous cell carcinomas, it is sometimes possible to depict the loss of DEJ, if hyperkeratosis is not too severe. Analogous to RCM, morphological criteria can be used for differentiation of melanocytic lesions, such as nevi and (in situ) melanomas. Evidence relating to melanoma includes disturbed epidermal architecture, pagetoid cells, or atypical melanocytic nests.^43^, ^44^, ^45^ Its use in diagnosis or therapy monitoring of infectious dermatoses, such as scabies, onychomycoses, or inflammatory dermatoses, such as psoriasis, and autoimmune blistering skin diseases is also feasible.^46^ In these areas, however, the technique has so far been used predominantly in the context of trials.

Typical characteristics of solid and superficial basal cell carcinomas are ovoid, hyporeflective tumor nests with dark rim. Infiltrative basal cell carcinomas are characterized by a shoal of fish‐like tumor strands. Squamous cell carcinomas usually present with hyperkeratosis and loss of the dermoepidermal junction. Melanocytic lesions can be interpreted analogous to the morphological criteria in RCM.

#### Case studies

**FIGURE 7:**
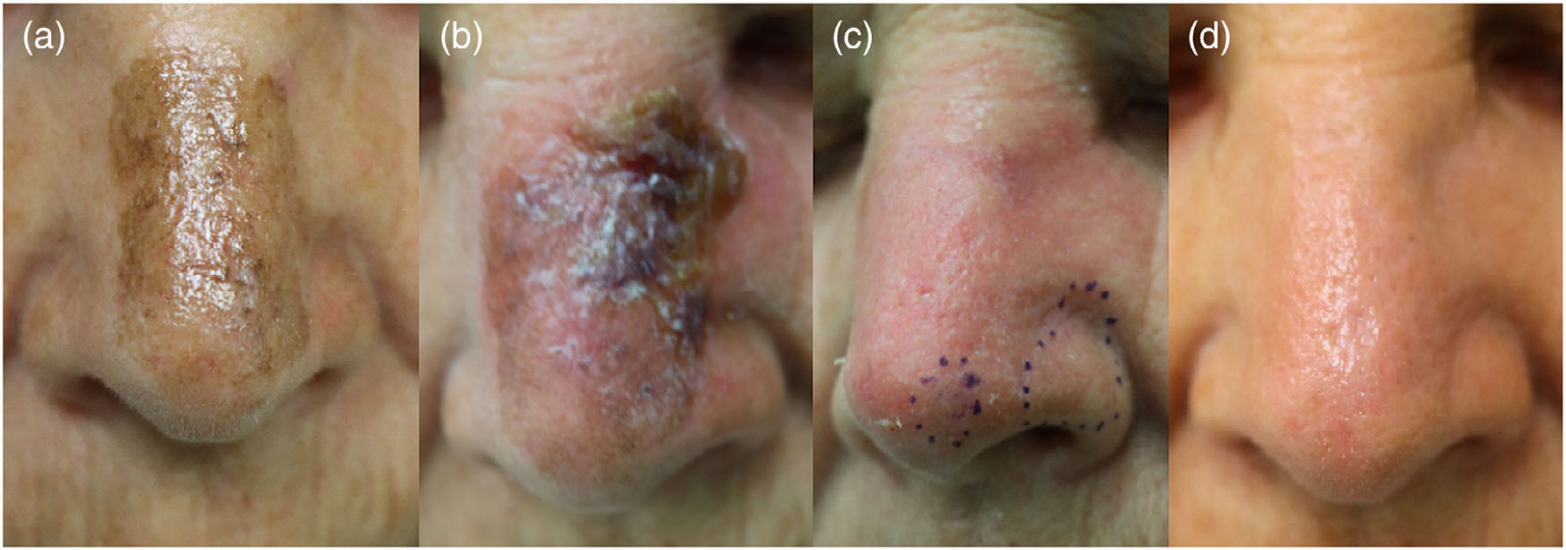
Clinical photographs of lentigo maligna of the nasal skin on topical treatment with imiquimod 5% cream. (a) Week 0. (b) Week 4. (c) Week 10: skin areas with residual lentigo maligna detected by line‐field confocal optical coherence tomography are indicated. (d) After 11 months.

**FIGURE 8:**
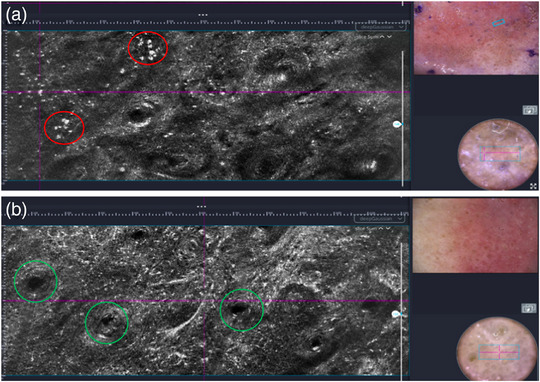
Lentigo maligna on topical treatment with imiquimod 5% cream at week 0 (top) and week 10 (bottom), visualized using line‐field confocal optical coherence tomography (LC‐OCT, deepLive™, DAMAE Medical, Paris, France; horizontal image size 1.2 × 0.5 mm) in horizontal mode. (a) Week 0: large, hyperreflective dendritic cells, particularly grouped around hair follicles, corresponding to atypical melanocytes (red circle), visible in the epidermis. The simultaneous dermoscopy of the lesion is shown on the right side of the image. (b) Week 10: dermoscopy no longer shows any pigment; atypical melanocytes are clearly rarefied but still present (green circle).

An elderly female patient presented with histologically confirmed lentigo maligna on the nasal skin. Given the size of the lesion, we opted together with the patient for a topical therapy with imiquimod 5% monitored by LC‐OCT. The therapy was initially prescribed once daily *off‐label*. In week 4 and week 6 (not shown), therapy had to be interrupted for several days due to strong local reactions. The clinical course is presented in Figure 7. An example of lentigo maligna in LC‐OCT is illustrated in the upper panel of Figure 8. With predominantly inconspicuous clinical and dermoscopic results, follow‐up with LC‐OCT was performed in week 10 (Figure 8, lower panel). At that time, areas with increased numbers of atypical melanocytes around the hair follicles were still detected on the left ala of the nose and the nasal tip. Selective topical treatment of the affected regions, again with imiquimod, was performed for 4 weeks. Subsequently, no lentigo maligna residuals were detected by LC‐OCT. Since then, the patient has been free of symptoms, and she is now only in clinical follow‐up.

#### Advantages and limitations

By combining the high resolution of RCM with a sufficient penetration depth and the vertical view of OCT, LC‐OCT distinguishes itself from the other imaging procedures. The fast measurement results in histology‐like findings. Of all existing noninvasive imaging methods, these findings resemble biopsy most closely. The tissue is visualized as bedside diagnosis in real time. With previous experience in histology or OCT, interpretation of the findings is easy to learn. Moreover, the diagnosis may be supported by additional use of artificial intelligence (AI). The software of the company DAMAE Medical is CE‐certified and can detect, for example, basal cell carcinomas with a sensitivity of 98% and a specificity of 81% (Figure 6c).^47^ The application spectrum of LC‐OCT combines the complete spectrum of RCM and OCT with some minor restrictions. So far, however, melanocytic lesions are differentiated with slightly higher resolution by RCM while assessment of the penetration depth of tumors with higher tumor thickness (> 0.5 mm) is better with OCT. The fast diagnostic workup may contribute to reduction of unnecessary biopsies especially in sensitive areas or in children. Moreover, use in, for example, basal cell carcinomas may simplify clinical routine with respect to presurgical demarcation of tumor extension, selection of treatment options based on subtype and tumor thickness, or assessment of local recurrence. Compared to RCM and OCT, the equipment costs are, however, slightly higher (Table 2).

**TABLE 2:** Overview of characteristics of the various non‐invasive imaging techniques: reflectance confocal microscopy (RCM), optical coherence tomography (OCT), and line‐field confocal optical coherence tomography (LC‐OCT).^48^

	RCM	OCT	LC‐OCT
Resolution	1–3 µm	7.5 µm	1–3 µm
Penetration depth/measurement depth	300 µm	Up to 1,500 µm	Up to 500 µm
Image view	Horizontal, video	Vertical, horizontal, 3D mode	Horizontal, vertical, 2D and 3D mode, video
Main indications	Melanocytic and epithelial tumors	Epithelial tumors	Melanocytic and epithelial tumors
AI‐enabled		Yes	Yes, CE‐certified
Special functions		Dynamic OCT	Conversion to *ex vivo* LC‐OCT possible
Instruments and manufacturers	VivaScope 1500/3000/Combo (VivaScope GmbH (Munich, Germany)	VivoSight Dx (VivoSight, Michelson Diagnostics, Kent, United Kingdom)	deepLive^TM^ (DAMAE Medical, Paris, France)
Instrument costs	From 70,000 Euro	From 85,000 Euro	From 150,000 Euro

*Abbr*.: CE, *Conformité Européenne*; RCM, reflectance confocal microscopy; LC‐OCT, *line‐field* confocal optical coherence tomography; OCT, optical coherence tomography

Given the resolution at the cellular level down to the mid‐dermis, *line‐field* confocal optical coherence tomography (LC‐OCT) enables the assessment of both epithelial and melanocytic lesions. However, the invasion depth of tumors can only be measured down to 0.5 mm.

### Outlook

Noninvasive imaging is an integral component of dermatological clinical routine and research. This is also reflected by the recently published S1 guideline for imaging diagnostics for skin diseases.^48^ Nevertheless, RCM, OCT, or LC‐OCT are not used ubiquitously as yet. Apart from financial aspects like equipment costs and remuneration, the widespread use in outpatient care is, among other factors, hampered by the effort to learn a new technique or operate new instruments (Table 2). Given that LC‐OCT combines the benefits of OCT and RCM in one instrument, this technique alone will be able to cover many clinical indications. The additional integration of AI supports not only the assessment of images but also the learning process of the physicians making the diagnosis. Measurement and diagnosis can be performed as bedside diagnosis in real time. The application spectrum is very wide and includes, apart from primary diagnosis and presurgical demarcation of skin tumors, also support in the selection of topical treatments by subtyping and determination of the penetration depth of cutaneous lesions, therapy monitoring of skin tumors, and assessment of recurrence. Moreover, the potential benefit of LC‐OCT in numerous infectious and inflammatory dermatoses has been demonstrated in trials. Although LC‐OCT has the widest spectrum of possible applications, it is slightly inferior to RCM and OCT in certain cases. Further technical developments may, however, remedy this situation in the future. The opportunity to assess the living skin in real time at the cellular level assisted by AI, if applicable, offers a vast potential for additional fields of application.

## CONFLICT OF INTEREST STATEMENT

None.

## CME Questions/Lernerfolgskontrolle

1. Welche der folgenden Aussagen zur Eindringtiefe und Auflösung der LC‐OCT ist zutreffend?
  - Im Vergleich zur optischen Kohärenztomographie weist die LC‐OCT eine deutlich höhere Eindringtiefe von bis zu 5 mm auf.
  - Die LC‐OCT weist eine ähnlich hohe Auflösung wie die konfokale Lasermikroskopie auf und kann deshalb auch zur Beurteilung von melanozytären Läsionen herangezogen werden.
  - Die Eindringtiefe der LC‐OCT ist deutlich geringer als die der konfokalen Lasermikroskopie.
  - Die Auflösung der LC‐OCT ist deutlich geringer als die der optischen Kohärenztomographie.
  - Die LC‐OCT weist eine höhere Eindringtiefe bei zudem höherer Auflösung im Vergleich zur optischen Kohärenztomographie auf.
2. Sie stellen bei einem Patienten klinisch den Verdacht auf mehrere superfizielle Basalzellkarzinome sowie eine melanozytäre Läsion unklarer Dignität. Welche der folgenden nichtinvasiven bildgebenden Techniken ist am ehesten geeignet, alle Läsionen zu beurteilen?
  - Auflichtmikroskopie
  - optische Kohärenztomographie
  - Line‐field konfokale optische Kohärenztomographie (LC‐OCT)
  - konfokale Lasermikroskopie
  - hochauflösender Ultraschall
3. Welche Aussage zur Beurteilung der Messungen der LC‐OCT trifft am ehesten zu?
  - Die Aufnahmen können KI‐assistiert beurteilt werden.
  - Die Aufnahmen können nur in Echtzeit analysiert werden. Eine Analyse im Nachgang ist nicht möglich.
  - Die Messungen müssen nach dem Speichern an spezialisierte Zentren zur Befundung versendet werden. Eine in‐vivo Befundung ist nicht möglich.
  - Das Erlernen der Beurteilung der LC‐OCT Messungen dauert in der Regel mehrere Jahre und sollte wenigen Experten vorbehalten sein.
  - Im Gegensatz zur konfokalen Lasermikroskopie können Blutgefäße nicht mithilfe der LC‐OCT analysiert werden.
4. Was sind typische morphologische Merkmale eines Basalzellkarzinoms vom infiltrativen Typ im LC‐OCT?
  - Fischschwarmartige Tumorstränge
  - polymorphe, helle atypische dendritische Zellen
  - Ovoide Tumornester mit peritumoraler Spaltbildung (clefting)
  - undulierende Hyperkeratosen
  - Pagetoide Zellen in der Epidermis
5. Welche Aussage zur optischen Kohärenztomographie (OCT) trifft am ehesten zu?
  - Die OCT ermöglicht ausschließlich horizontale Bildaufnahmen der Haut.
  - Die maximale Eindringtiefe der OCT liegt bei etwa 300 µm.
  - Die OCT eignet sich besonders zur Beurteilung der Tumordicke und lateralen Ausdehnung bei Basalzellkarzinomen.
  - Die OCT hat eine höhere Auflösung als die konfokale Lasermikroskopie.
  - Mit der OCT lassen sich atypische Melanozyten auf zellulärer Ebene differenzieren.
6. Welches der folgenden Merkmale ist nicht typisch für ein noduläres Basalzellkarzinom (nBCC) in der OCT?
  - Große, runde hyporeflektive Tumornester
  - Starke Verbindung zur Epidermis mit Unterbrechung der der dermoepidermalen Junktionszone.
  - Peritumorale Spaltbildung mit hyperreflektivem Stroma
  - Teilweise Verbindung zur Epidermis
  - Knotiges Wachstumsmuster
7. Welches Bildmuster ist typisch für gesunde Haut in der konfokalen Lasermikroskopie?
  - Diffuse, helle Areale ohne klare Struktur
  - Dunkle unregelmäßige Zellnester mit zentraler Aufhellung
  - Honigwabenmuster im Stratum spinosum
  - Fischschwarmartige Tumorstrukturen
  - Clefting mit Tumorzapfen
8. Welche der folgenden Aussagen zur KLM trifft am ehesten zu?
  - Die KLM erreicht eine Eindringtiefe von bis zu 2 mm.
  - Die KLM kann auch tiefe Tumorkomponenten invasiver Basalzellkarzinome vollständig darstellen.
  - Die KLM eignet sich besonders zur Differenzierung von melanozytären Läsionen.
  - Die KLM zeigt grundsätzlich keine Darstellung von Melanin.
  - Die KLM ersetzt in jedem Fall die histologische Diagnostik.
9. Was ist ein wesentlicher Unterschied zwischen der konfokalen Lasermikroskopie (KLM) und der optischen Kohärenztomographie (OCT)?
  - Die KLM hat eine höhere Eindringtiefe als die OCT.
  - Die OCT ermöglicht eine bessere Differenzierung von pigmentierten melanozytären Läsionen.
  - Die KLM stellt Zellen detaillierter auf zellulärer Ebene dar, die OCT hingegen besser tiefere Gewebestrukturen.
  - Beide Methoden verwenden identische Lichtquellen und Scanverfahren.
  - Die KLM wird ausschließlich für entzündliche Dermatosen eingesetzt.
10. Welche Aussage zur dynamischen optischen Kohärenztomographie (D‐OCT) trifft am ehesten zu?
  - Die D‐OCT eignet sich ausschließlich zur Beurteilung von pigmentierten melanozytären Läsionen.
  - Die D‐OCT ermöglicht durch die Analyse bewegter Teilchen eine Darstellung der Mikrovaskularisation in der Haut.
  - Die D‐OCT zeigt eine höhere laterale Auflösung als die konfokale Lasermikroskopie.
  - Die D‐OCT ersetzt die histopathologische Diagnostik bei malignen Tumoren vollständig.
  - Die D‐OCT ist auf die Visualisierung des Stratum corneum beschränkt.

Liebe Leserinnen und Leser, der Einsendeschluss an die DDA für diese Ausgabe ist der 31. Januar 2026.

Die richtige Lösung zum Thema Vitiligo in Heft 08/2025 ist: 1b, 2c, 3d, 4f, 5b, 6b, 7d, 8b, 9e, 10a

Bitte verwenden Sie für Ihre Einsendung das aktuelle Formblatt auf der folgenden Seite oder aber geben Sie Ihre Lösung online unter http://jddg.akademie-dda.de ein.

## References

[1] Schmitz L , Reinhold U , Bierhoff E , Dirschka T . Optical coherence tomography: its role in daily dermatological practice. J Dtsch Dermatol Ges. 2013;11:499‐507. 23565622 10.1111/ddg.12073

[2] Huang D , Swanson EA , Lin CP , et al. Optical coherence tomography. Science. 1991;254:1178‐1181. 1957169 10.1126/science.1957169 PMC4638169

[3] Ogien J , Tavernier C , Fischman S , Dubois A . Line‐field confocal optical coherence tomography (LC‐OCT): principles and practical use. Ital J Dermatol Venerol. 2023;158:171‐179. 37278495 10.23736/S2784-8671.23.07613-2

[4] Branzan AL , Landthaler M , Szeimies R‐M . In vivo confocal scanning laser microscopy in dermatology. Lasers Med Sci. 2007;22:73‐82. 17115235 10.1007/s10103-006-0416-8

[5] González S , Gilaberte‐Calzada Y . In vivo reflectance‐mode confocal microscopy in clinical dermatology and cosmetology. Int J Cosmet Sci. 2008;30:117. 10.1111/j.1468-2494.2008.00406.x 18377626

[6] González S , Swindells K , Rajadhyaksha M , Torres A . Changing paradigms in dermatology: confocal microscopy in clinical and surgical dermatology. Clin Dermatol. 2003;21:359‐369. 14678715 10.1016/j.clindermatol.2003.08.007

[7] Levine A , Markowitz O . Introduction to reflectance confocal microscopy and its use in clinical practice. JAAD Case Rep. 2018;4:1014‐1023. 30456275 10.1016/j.jdcr.2018.09.019 PMC6232695

[8] Farnetani F , Scope A , Braun RP , et al. Skin Cancer Diagnosis With Reflectance Confocal Microscopy: Reproducibility of Feature Recognition and Accuracy of Diagnosis. JAMA Dermatol. 2015;151:1075‐1080. 25993262 10.1001/jamadermatol.2015.0810

[9] Pellacani G , Guitera P , Longo C , et al. The impact of in vivo reflectance confocal microscopy for the diagnostic accuracy of melanoma and equivocal melanocytic lesions. J Invest Dermatol. 2007;127:2759‐2765. 17657243 10.1038/sj.jid.5700993

[10] Couty E , Tognetti L , Labeille B , et al. In vivo reflectance confocal microscopy combined with the “spaghetti technique” for the identification of surgical margins of lentigo maligna: experience in 70 patients. J Eur Acad Dermatol Venereol. 2018;32:e366‐e368. 29573292 10.1111/jdv.14947

[11] Ardigo M , Malizewsky I , Dell'anna ML , et al. Preliminary evaluation of vitiligo using in vivo reflectance confocal microscopy. J Eur Acad Dermatol Venereol. 2007;21:1344‐1350. 17958840 10.1111/j.1468-3083.2007.02275.x

[12] Cortelazzi C , Pellacani G , Raposio E , Di Nuzzo S . Vitiligo management: combination of surgical treatment and phototherapy under reflectance confocal microscopy monitoring. Eur Rev Med Pharmacol Sci. 2020;24:7366‐7371. 32706075 10.26355/eurrev_202007_21904

[13] Wang H‐F , Wang C‐Y , Zhou X‐F , et al. A New Assessment Method of Vitiligo by Combination of Dermoscopy and Reflectance Confocal Microscopy. Clin Cosmet Investig Dermatol. 2023;16:3615‐3623. 10.2147/CCID.S432169 PMC10740724 38144155

[14] Pellacani G , Pepe P , Casari A , Longo C . Reflectance confocal microscopy as a second‐level examination in skin oncology improves diagnostic accuracy and saves unnecessary excisions: a longitudinal prospective study. Br J Dermatol. 2014;171:1044‐1051. 24891083 10.1111/bjd.13148

[15] Witkowski AM , Łudzik J , Arginelli F , et al. Improving diagnostic sensitivity of combined dermoscopy and reflectance confocal microscopy imaging through double reader concordance evaluation in telemedicine settings: A retrospective study of 1000 equivocal cases. PLoS One. 2017;12:e0187748. Available from: URL:https://journals.plos.org/plosone/article?id=10.1371/journal.pone.0187748 [Last accessed on August 11, 2025]. 29121636 10.1371/journal.pone.0187748 PMC5679638

[16] Segura S , Puig S , Carrera C , Palou J , Malvehy J . Development of a two‐step method for the diagnosis of melanoma by reflectance confocal microscopy. J Am Acad Dermatol. 2009;61:216‐229. 19406506 10.1016/j.jaad.2009.02.014

[17] Olsen J , Holmes J , Jemec GB . Advances in optical coherence tomography in dermatology‐a review. J Biomed Opt. 2018;23:1‐10. 10.1117/1.JBO.23.4.040901 29701018

[18] Schuh S , Holmes J , Ulrich M , et al. Imaging Blood Vessel Morphology in Skin: Dynamic Optical Coherence Tomography as a Novel Potential Diagnostic Tool in Dermatology. Dermatol Ther (Heidelb). 2017;7:187‐202. 28258554 10.1007/s13555-017-0175-4 PMC5453917

[19] Welzel J , Schuh S . Nichtinvasive Diagnostik in der Dermatologie. J Dtsch Dermatol Ges. 2017;15:999‐1017. 10.1111/ddg.13347_g 28976095

[20] Lang BM , Balermpas P , Bauer A , et al. S2k‐Leitlinie Basalzellkarzinom der Haut ‐ Teil 2: Therapie, Prävention und Nachsorge. J Dtsch Dermatol Ges. 2019;17:214‐231. 10.1111/ddg.13755_g 30762951

[21] Welzel J , Schuh S . [Optical coherence tomography for skin pathologies]. Ophthalmologe. 2018;115:524‐527. 29774372 10.1007/s00347-018-0718-9

[22] Parashar K , Torres AE , Boothby‐Shoemaker W , et al. Imaging technologies for presurgical margin assessment of basal cell carcinoma. J Am Acad Dermatol. 2023;88:144‐151. 34793927 10.1016/j.jaad.2021.11.010

[23] Alawi SA , Kuck M , Wahrlich C , et al. Optical coherence tomography for presurgical margin assessment of non‐melanoma skin cancer – a practical approach. Exp Dermatol. 2013;22:547‐551. 23879814 10.1111/exd.12196

[24] Vélez González JJ , Berger M , Schiele S , et al. Dynamic optical coherence tomography of chronic venous ulcers. J Eur Acad Dermatol Venereol. 2024;38:223‐231. 37669869 10.1111/jdv.19496

[25] Mogensen M , Thrane L , Jørgensen TM , et al. OCT imaging of skin cancer and other dermatological diseases. J Biophotonics. 2009;2:442‐451. 19557752 10.1002/jbio.200910020

[26] Friis KBE , Themstrup L , Jemec GBE . Optical coherence tomography in the diagnosis of actinic keratosis‐A systematic review. Photodiagnosis Photodyn Ther. 2017;18:98‐104. 28188920 10.1016/j.pdpdt.2017.02.003

[27] Fuchs CSK , Ortner VK , Mogensen M , et al. 2021 international consensus statement on optical coherence tomography for basal cell carcinoma: image characteristics, terminology and educational needs. J Eur Acad Dermatol Venereol. 2022;36:772‐778. 35141952 10.1111/jdv.17969

[28] Olsen J , Themstrup L , De Carvalho N , et al. Diagnostic accuracy of optical coherence tomography in actinic keratosis and basal cell carcinoma. Photodiagnosis Photodyn Ther. 2016;16:44‐49. 27519350 10.1016/j.pdpdt.2016.08.004

[29] Holm KBE , Nielsen LJ , Lock‐Andersen J , et al. Optical coherence tomography for presurgical delineation of basal cell carcinomas on the face‐A comparison with histopathology. J Cutan Pathol. 2023;50:441‐9. 36794511 10.1111/cup.14412

[30] Markowitz O , Schwartz M , Feldman E , et al. Evaluation of Optical Coherence Tomography as a Means of Identifying Earlier Stage Basal Cell Carcinomas while Reducing the Use of Diagnostic Biopsy. J Clin Aesthet Dermatol. 2015;8:14‐20. PMC4633207 26557214

[31] Akella SS , Lee J , May JR , et al. Using optical coherence tomography to optimize Mohs micrographic surgery. Sci Rep. 2024;14:8900. 38632358 10.1038/s41598-024-53457-7 PMC11024158

[32] Wolswijk T , Adan F , Nelemans PJ , et al. Optical coherence tomography for diagnosing recurrent or residual basal cell carcinoma after topical treatment: A diagnostic cohort study. J Am Acad Dermatol. 2023;89:728‐733. 37391069 10.1016/j.jaad.2023.06.033

[33] de Giorgi V , Stante M , Massi D , et al. Possible histopathologic correlates of dermoscopic features in pigmented melanocytic lesions identified by means of optical coherence tomography. Exp Dermatol. 2005;14:56‐59. 15660920 10.1111/j.0906-6705.2005.00229.x

[34] Perwein MKE , Welzel J , De Carvalho N , et al. Dynamic Optical Coherence Tomography: A Non‐Invasive Imaging Tool for the Distinction of Nevi and Melanomas. Cancers (Basel). 2022;15:20. 36612016 10.3390/cancers15010020 PMC9817967

[35] Sahu A , Yélamos O , Iftimia N , et al. Evaluation of a Combined Reflectance Confocal Microscopy‐Optical Coherence Tomography Device for Detection and Depth Assessment of Basal Cell Carcinoma. JAMA Dermatol. 2018;154:1175‐1183. 30140851 10.1001/jamadermatol.2018.2446 PMC6179925

[36] Lang BM , Balermpas P , Bauer A , et al. S2k‐Leitlinie Basalzellkarzinom der Haut ‐ Teil 1: Epidemiologie, Genetik und Diagnostik. J Dtsch Dermatol Ges. 2019;17:94‐104. 10.1111/ddg.13733_g 30615280

[37] Dubois A , Levecq O , Azimani H , et al. Line‐field confocal optical coherence tomography for high‐resolution noninvasive imaging of skin tumors. J Biomed Opt. 2018;23:1‐9. 10.1117/1.JBO.23.10.106007 30353716

[38] Latriglia F , Ogien J , Tavernier C , et al. Line‐Field Confocal Optical Coherence Tomography (LC‐OCT) for Skin Imaging in Dermatology. Life (Basel.) 2023;13:2268. 38137869 10.3390/life13122268 PMC10744435

[39] Ruini C , Schuh S , Gust C , et al. Line‐field optical coherence tomography: in vivo diagnosis of basal cell carcinoma subtypes compared with histopathology. Clin Exp Dermatol. 2021;46:1471‐1481. 34047380 10.1111/ced.14762

[40] Suppa M , Fontaine M , Dejonckheere G , et al. Line‐field confocal optical coherence tomography of basal cell carcinoma: a descriptive study. J Eur Acad Dermatol Venereol 2021;35:1099‐1110. 33398911 10.1111/jdv.17078

[41] Verzì AE , Micali G , Lacarrubba F . Line‐Field Confocal Optical Coherence Tomography May Enhance Monitoring of Superficial Basal Cell Carcinoma Treated with Imiquimod 5 % Cream: A Pilot Study. Cancers (Basel). 2021;13:4913. 34638396 10.3390/cancers13194913 PMC8507996

[42] Kranz S , Brunnmeier G , Yilmaz P , et al. Optical coherence tomography‐guided Nd:YAG laser treatment and follow‐up of basal cell carcinoma. Lasers Surg Med. 2023;55:257‐267. 36740365 10.1002/lsm.23638

[43] Ruini C , Schuh S , Gust C , et al. In‐Vivo LC‐OCT Evaluation of the Downward Proliferation Pattern of Keratinocytes in Actinic Keratosis in Comparison with Histology: First Impressions from a Pilot Study. Cancers (Basel). 2021;13:2856. 34201052 10.3390/cancers13122856 PMC8228287

[44] Verzì AE , Broggi G , Caltabiano R , et al. Line‐field confocal optical coherence tomography of lentigo maligna with horizontal and vertical histopathologic correlations. J Cutan Pathol. 2023;50:118‐122. 36056910 10.1111/cup.14321 PMC10087826

[45] Perez‐Anker J , Soglia S , Lenoir C , et al. Criteria for melanocytic lesions in LC‐OCT. J Eur Acad Dermatol Venereol. 2024;38:2005‐2016. 38727525 10.1111/jdv.20079

[46] Eijkenboom QL , Daxenberger F , Gust C , et al. Line‐field confocal optical coherence tomography, a novel non‐invasive tool for the diagnosis of onychomycosis. J Dtsch Dermatol Ges. 2024;22:367‐375. 38279541 10.1111/ddg.15310

[47] Gust C , Schuh S , Welzel J , et al. Line‐Field Confocal Optical Coherence Tomography Increases the Diagnostic Accuracy and Confidence for Basal Cell Carcinoma in Equivocal Lesions: A Prospective Study. Cancers (Basel). 2022;14:1082. 35205830 10.3390/cancers14041082 PMC8870684

[48] S1‐Leitline Bildgebende Diagnostik von Hauterkrankungen AWMF‐Register‐Nr.:013‐076, 2024. Available from: URL: https://register.awmf.org/assets/guidelines/013‐076l_S1_Bildgebende‐Diagnostik‐von‐Hauterkrankungen_2024‐11.pdf [Last accessed on August 11, 2025].

